# Ocular and periocular radiation toxicity in dogs treated for sinonasal tumors: A critical review

**DOI:** 10.1111/vop.12761

**Published:** 2020-04-12

**Authors:** Friederike Wolf, Valeria S. Meier, Simon A. Pot, Carla Rohrer Bley

**Affiliations:** ^1^ Division of Radiation Oncology Vetsuisse Faculty University of Zurich Zurich Switzerland; ^2^ Department of Physics University of Zurich Zurich Switzerland; ^3^ Ophthalmology Section Vetsuisse Faculty University of Zurich Zurich Switzerland

**Keywords:** canine, radiotherapy, side effects on eyes, sinonasal tumors, veterinary

## Abstract

Visual impairment from radiation‐induced damage can be painful, disabling, and reduces the patient's quality of life. Ocular tissue damage can result from the proximity of ocular organs at risk to irradiated sinonasal target volumes. As toxicity depends on the radiation dose delivered to a certain volume, dose‐volume constraints for organs at risk should ideally be known during treatment planning in order to reduce toxicity. Herein, we summarize published ocular toxicity data of dogs irradiated for sinonasal tumors from 36 publications (1976‐2018). In particular, we tried to extract a dose guideline for a clinically acceptable rate of ocular toxicity. The side effects to ocular and periocular tissues were reported in 26/36 studies (72%) and graded according to scoring systems (10/26; 39%). With most scoring systems, however, toxicities of different ocular and periocular tissues are summed into one score. Further, the scores were mostly applied in retrospect and lack volume‐ and dose‐data. This incomplete information reflects the crux of the matter for radiation dose tolerance in canine ocular tissues: The published information of the last three decades does not allow formulating dose‐volume guidelines. As a start, we can only state that a mean dose of 39 Gy (given in 10 x 4.2 Gy fractions) will lead to loss of vision by one or both eyes, while mean doses of <30 Gy seem to preserve functionality. With a future goal to define tolerated doses and volumes of ocular and periocular tissues at risk, we propose the use of combined ocular toxicity scoring systems.

## 1 INTRODUCTION

Is it still necessary to prepare owners for severe ocular side effects in dogs treated with radiation therapy for sinonasal tumors? Advances in the technology of radiation‐planning and dose delivery, such as intensity‐modulated radiation therapy (IMRT), can dramatically decrease acute radiation side effects. IMRT allows the dose to be more accurately focused onto the tumor, while sparing surrounding normal tissues. These surrounding tissue tolerances define the limit (constraint goals) determined at the beginning of the treatment planning process and are used to optimize dose. In order to find dose guidelines as constraints for a clinically acceptable rate of ocular toxicity, we screened the current literature for ocular toxicity data and corresponding radiation doses of dogs irradiated for sinonasal tumors.

For each disease entity treated with radiation therapy, a prescribed dose protocol competes between tumor destruction and the preservation of healthy, surrounding tissues. Treatment of sinonasal tumors with radiation therapy has a long‐standing history in veterinary radiation therapy. Due to the close proximity of ocular tissues to sinonasal target volumes, ocular tissue damage can occur. Preservation of organ function, however, is an important aim during the planning process. Prior to the use of modern linear accelerators in veterinary medicine, moderate to severe damage to ocular structures was common; this resulted in occasionally painful early and late toxicities often leading to unilateral loss of vision and/ or loss of the eye.^1^, ^2^, ^3^, ^4^, ^5^, ^6^, ^7^, ^8^

We still do not know accurate tolerance doses for ocular structures in veterinary medicine. Human radiation oncologists on the other hand have collected a vast number of acceptable toxicity‐dose levels for various normal tissues. With the intention to summarize and discuss normal tissue toxicity, the Quantitative Analyses of Normal Tissue Effects in the Clinic (QUANTEC) reports were created. Those reports contain a series of publications on quantitative analyses of normal tissue effects in the clinic.^9^, ^10^ As a result, dose‐volume constraint guidelines for the optic nerve and chiasm have been provided.^10^ To create these reports, the toxicity‐dose levels were derived from the conventional photon radiation‐planning era in human radiation oncology and serve as simple approach for toxicity tolerance in clinical treatment planning. These dose levels consist of dose‐volume numbers and are now often used as constraints or dose limits in the complex IMRT‐planning process. In order to create such reports and find acceptable dose levels for clinical use, standardized continuous scoring and documentation of normal tissue toxicity is necessary. Such scoring systems exist for different organs at risk, for example, the Toxicity Criteria of the Radiation Therapy Oncology Group (RTOG), and include objective criteria provided by the medical care team, mostly from radiation oncology.^11^ For late toxicity, an additional system is commonly used, which includes criteria from four categories (1‐ Subjective criteria from the patient, 2‐ Objective criteria, 3‐ Management criteria, and 4‐ Analytic criteria) known as the **LENT‐SOMA** scales: **L**ate **E**ffect of **N**ormal **T**issue—**S**ubjective, **O**bjective, **M**anagement, **A**nalytic.^12^ The LENT‐SOMA classification has the advantage of using a more detailed terminology for the specific side effects (other than the terms “mild”, “moderate”, and “severe” as used in the RTOG criteria) and can therefore also be used to score side effects from other oncologic treatments such as surgery or chemotherapy. It also includes management and analytic (eg, measurable) criteria in a systematic way. An adapted version of the RTOG criteria is commonly used in veterinary medicine: the Toxicity Criteria of the Veterinary Radiation Therapy Oncology Group (VRTOG), which provides scores for both early and late toxicity.^13^

While toxicity is often described in the veterinary radiation oncology literature, use of toxicity scoring systems, including evaluations at fixed time points, is neither systematically evaluated nor reported. Furthermore, the categories of the VRTOG or RTOG scoring systems often sum up different parts of an organ (eg, cornea, conjunctiva, and lens) in one category (eg, “eye”). This creates problems when retrospectively determining which specific part of the organ was involved to which degree. To complicate the matter, fractionation schedules and prescribed doses, both important factors to calculate or estimate the risk of side effects, often vary between institutions.

In this review, we summarize available information on ocular and periocular toxicity that can occur after radiation therapy for sinonasal tumors in dogs. With a future goal to define the tolerated doses and volumes of ocular and periocular tissues at risk, we screened the publications of canine sinonasal tumors for information on toxicity after radiation therapy and propose the use of more detailed ocular toxicity scoring systems.

## 2 CLINICAL SIGNIFICANCE OF OCULAR TOXICITY

**Figure 1:**
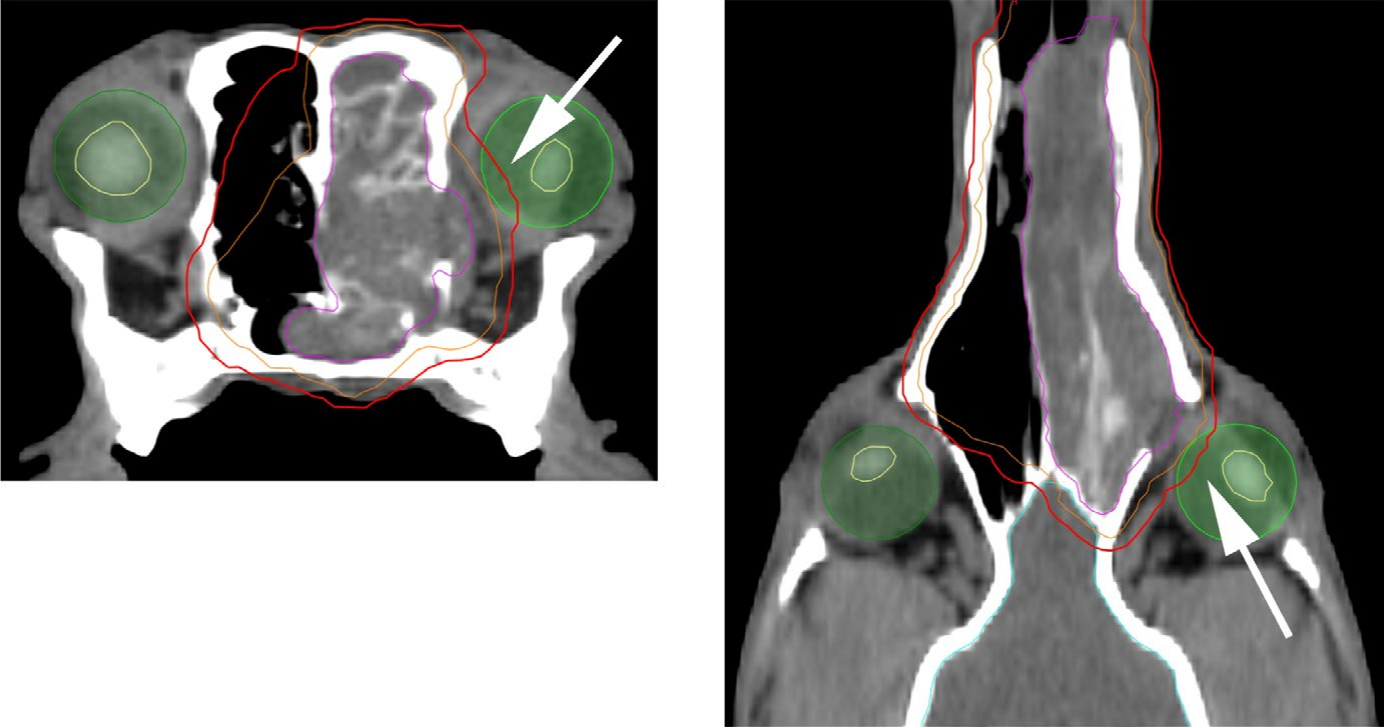
Planning‐CT of a dog with sinonasal tumor (lefthand side: transversal image at the level of the eyes, righthand side: dorsal image at the level of the eyes). Bright green: ipsilateral eye with ocular lens (yellow), pale green: contralateral eye with ocular lens (yellow). Blue: brain. Pink contour: visible extent of the malignant growth = gross tumor volume (GTV), orange contour: additional margin that includes suspected microscopic disease = clinical tumor volume (CTV), red contour: planning target volume (PTV), this volume may include parts of the eye(s) (white arrow) since it takes the sum of geometrical variations and inaccuracies into consideration

The complex anatomical structure of the sinonasal cavity and the infiltrative nature of the tumors make the treatment of nasal and paranasal sinus tumors difficult.^14^, ^15^ Radiation therapy is the current standard of care, but optimal protocols have not been defined.^6^, ^16^, ^17^ Radiation therapy improves clinical signs, delays time to progression and prolongs overall survival time. ^5^, ^6^, ^18^, ^19^, ^20^, ^21^, ^22^, ^23^, ^24^ A definitive cure, however, is rarely obtained.^25^, ^26^ Because treatment usually fails locally, veterinary radiation oncologists are motivated to try out different radiation protocols, fraction sizes, and prescribed doses (Table 1). The anatomical configuration of the dog's skull enhances the challenge, as the radiation sensitive ocular structures including tear gland, corneal endothelium, lens, and retina often lie very close to or even in the treated volume.^27^ The visible extent of the malignant growth is represented by the gross tumor volume (GTV) and the clinical target volume (CTV) represents the GTV and an additional margin that includes additional microscopic disease. These first two volumes are based on the observed size, type, and known locally malignant/infiltrative behavior of the tumor and do not encompass the eyes (Figure 1). The planning target volume (PTV), however, may include parts of the eye(s) since it takes the sum of geometrical variations and inaccuracies into consideration to ensure that the prescribed dose is actually absorbed in the CTV (Figure 1). Additional organs surrounding the target are defined by the radiation oncologist, contoured and integrated into the treatment planning, in order to quantify the radiation dose to these tissues. These organs at risk (OAR) such as the globe and lenses are slightly mobile and changes in position during treatment can result in a difference between planned and delivered dose.^27^ As shown in one institution, the definition of the volumes for sinonasal tumors in dogs can represent a challenge to radiation oncologists: Inter‐operator contouring variability was found to occur for the target volumes but also for organs at risk.^28^ Depending on the radiation techniques, the machine's and institute's treatment accuracy as well as doses used, a wide spectrum of side effects is possible. These side effects may influence the patients’ comfort and vision over a broad range: Dogs may experience mild side effects only or may suffer from painful corneal ulcers or even lose vision permanently. Visual impairment from radiation‐induced damage is rarely life‐threatening, but disabling and reduces the patient's quality of life. Early radiation toxicity in ocular and periocular structures may include painful inflammatory side effects such as severe keratitis and corneal ulcers, whereas late toxicities may include chronic keratoconjunctivitis sicca (KCS), cataracts and/or vascular injury, which can contribute to the development of retinal hemorrhages, glaucoma and optic neuropathy.

**Table 1:** Summary of 36 published reports on radiation treatment of sinonasal tumors in dogs

Investigator	Dogs	Equipment Energy Technique	Fraction #	Fraction size ^13^ [Gy]	Prescribed dose [Gy]	Overall treatment time [d]	Toxicity described	Acute toxicities [%], refers to the number of patients with the respective toxicity						Late toxicities [%], refers to the number of patients with the respective toxicity					Dose on eye Median/mean
								VRTOG						VRTOG					
								0	1	2	3			0	1	2	3		
Soukup et al, (2018)^41^	9	Linac 6MV SIB‐IMRT	10	4.2 plus 20% SIB to GTV	42	12	Yes	6/9 (67%)	3/9 (33%)	0	0			na	na	na	na		4.1‐38.9 Gy D50% (median dose): 14.5‐17.6
Gieger et al, (2018)^85^	29	Linac‐based SRT 6MV	3	10	30	3	Yes	nd	nd	nd	nd			nd	nd	nd	nd		nd
Kubicek et al, (2016)^20^	57	Linac‐based SRS 6 MV	1	12‐28 to GTV	mean GTV: 30	1	Yes		1/57 (2%)							8/57 (14%)			nd
Bowles et al, (2016)^16^	16	Linac 4 or 6 MV 3DCRT, surgery	16‐18	3	48‐54	16‐18	Yes		2/15 (13%)					nd	nd	nd			nd
Cancedda et al, (2015)^17^	24	Linac 6 MV 3CRT	5 or 10	6 or 3	30	17 or 10	Yes			5/24 (21%)					2/24 (8%)				nd
Fujiwara et al, (2013)^54^	38	Linac 4MV 3DCRT	2‐4	6‐10	16‐32	14 or 30	Yes		26/36 (72%)^a^	5/36 (14%)^a^					10/30 (33%)^a^	11/30 (37%)^a^			nd
Sones et al, (2013)^24^	86	Linac nd nd	5‐20	3‐8	20‐60	5‐20	Yes								10/?^a^	7/?^a^			
Pinard et al, (2012)^8^	37	Cobalt‐60 teletherapy 2D‐RT (nd)	3‐24	2.5‐8	18‐60	20‐34	Yes		37/37 (100%)^b^	24/37 (71%)^b^	9/37 (27%)				7/37 (21%)	9/37 (24%)	2/37 (6%)		nd
Maruo et al, (2011)^86^	63	Linac 4 MV 3DCRT	4	5‐10	32 median	30	Yes		13/63 (21%)					nd	nd	nd			nd
Hunley et al, (2010)^43^	12	Linac 6 MV IMRT	18‐21	3	54‐63	42‐49	Yes		1/12 (8%)	4/12 (33%)	1/12 (8%)					1/12 (8%)	1/12 (8%)		nd
Lawrence et al, (2010)^22^	31	Tomotherapy 6 MV IMRT	10	4.2	42	12	Yes	Different scoring system used, mostly mild side effects (in 7/31 (23%)), no blind eyes						Mostly mild side effects in 8/31 (26%), no blind eyes					Mean 12Gy
Lawrence et al, (2010)^22^	36	Cobalt‐60 teletherapy 2D‐RT (historical control group)	10	4.2	42	12	Yes	Moderate to severe side effects (in 35/36 (97%)), loss of vision (one or both eyes) in 20 (56%), unclear if acute or late						Symptomatic (moderate to severe) side effects (in 23/36 (64%)), loss of vision (one or both eyes) in 20 (56%), unclear if acute or late					Mean 33.6, mean dose to 26 blind eyes: 39; mean dose to 23 sighted eyes 29.9
Adams et al, (2009)^87^	94	Cobalt‐60 teletherapy 60 2D‐RT (nd)	10	4.2	42	12	No												
		Linac 4 MV	17‐19	3	51‐57	22‐24													
Buchholz et al, (2009)^88^	38	Linac 6 MV 3DCRT	3‐10	3‐8	24‐32	17‐28	Yes		7/38 (18%)^a^					nd	nd	nd			nd
Mayer‐Stankeova et al, (2009)^68^	30	Protons pencil beam spot	10‐17	3.5	24.5‐59.5 (escalation)	17‐28	Yes			RTOG 18/18 (100%)^a^						RTOG 12/12 (100%)^a^			≥90% of prescribed dose in one eye (n = 18)
Gieger et al, (2008)^55^	48	Linac Cobalt‐60 teletherapy 60 2D‐RT	2‐5	4‐10	16‐40	21‐28	Yes		14/40 (35%)^a^								5/39 (13%)^a^		39/48 (81%) at least one eye was irradiated
Adams et al, (2005)^6^	53	Cobalt‐60 teletherapy 60 2D‐RT (13 surg)	10	4.2	42	12	Yes	nd	nd	nd							15/53 (28%)^a^		nd
Lana et al, (2004)^56^	51	Linac 6 MV 2D‐RT + cisplatin	18	3	50‐54	18	Yes	12/51 (23%)	11/51 (22%)	20/51 (39%)		8/51 (16%)				14/39 (36%)			nd
LeBlanc et al, (2004)^62^	15	Linac 4MV Cobalt‐60 teletherapy 60 + gemcitabine	17	3‐3.2	45‐54	17	Yes	5/15 (33%)	3/15 (20%)	3/15 (20%)		2/15 (13%)		na	na	na	na		nd
Nadeau et al, (2004)^57^	31	Cobalt‐60 teletherapy 60 2D‐RT + cisplatin(18)	12	4‐4.2	40‐50	28	yes	6/31 (19%)	3/31 (10%)	14/31 (45%)		8/31 (26%)			5/31 (16%)	1/31 (3%)	4/31 (13%)		nd
Correa et al, (2003)^90^	6	Cobalt‐60 teletherapy 60 2D‐RT	18‐21	3	54‐63	18	Yes			3/? ^a^									
Green et al, (2002)^91^	24	Cobalt‐60 teletherapy 60 2D‐RT	4	8	32	4	Yes	nd	nd	nd		nd		nd	nd	nd	nd		nd
Mellanby et al, (2002)^92^	56	Linac 4 MeV 2D‐RT	4	9	36	4	Yes			7/56 (13%)^a^							1/23 (4%)^a^		nd
Northrup et al, (2001)^29^	42	Orthovoltage + surgery	12	4	48	22‐44	Yes	RTOG 2/42 (5%)	RTOG 13/42 (31%)	RTOG 18/42 (43%)		RTOG 7/42 (17%)	RTOG 0/42 (0%)	RTOG 7/42 (17%)	RTOG 13/42 (31%)	RTOG 3/42 (7%)	RTOG 1/42 (2%)	RTOG 2/42 (5%)	All dogs had at least one eye in radiation field
LaDue et al, (1999)^21^	130	Cobalt‐60 teletherapy 60 2D‐RT	18‐19	3	54‐57	30	Yes	17/109 (16%)^a^	62/109 (57%)^a^	30/109 (28%)^a^						42/109 (39%)^a^			nd
Adams et al, (1998)^5^	21	Cobalt‐60 teletherapy 60 2D‐RT	9‐10	4.2	42	11‐13	Yes		20/21 (95%)^a^	1/21 (5%)^a^					13/19 (68%)^a^	12/19 (63%)^a^	6/15 (40%)^a^		nd
Henry et al, (1998)^25^	56	Cobalt‐60 teletherapy 60 2D‐RT	10	1.7‐4.8	17‐48		No												
Theon et al, (1993)^30^	77	Cobalt‐60 teletherapy 60 2D‐RT	12	4	48	28	Yes			(44%)^a^		(4%) ^a^				(45%)^a^	4/77 (5%)^a^		51% 1 eye, 49% 2 eyes in field
Thrall et al, (1993)^32^	24	Cobalt‐60 teletherapy 60 2D‐RT	8‐45	1.5‐4.5	36‐67.5	28	Yes		7/?^a^										
Jamieson et al, (1991)^33^	27	Cobalt‐60 teletherapy 60 2D‐RT	8‐42		36‐67.5		Yes		10/37 (27%)^a^	7/37 (19%)^a^		5/37 (13%)^a^			21/37 (57%)^a^	13/37 (35%)^a^			25/27 (93%): part or all of 2 eyes in field
McEntee et al, (1991)^69^	27	Cobalt‐60 teletherapy −60 2D‐RT	10‐12	3.5‐5.4	42‐54	28	Yes		18/27 (67%)^a^							13/27 (48%)^a^			often possible to exclude one eye
Ching et al, (1990)^7^	37	Linac 6 MV		1.5‐ 4.5	36‐67.5	28	Yes			RTOG6/7 (86%)^a^							RTOG 4/9 (44%)^a^		90% of dose ipsilat. Eye 35% contralat. eye
Adams et al, (1987)^18^	67	Cobalt‐60 teletherapy 60 Linac 6MV Cesium Orthovoltage	10	3.2‐5.2	36‐52	22	Yes			1/?^a^					1/?^a^				
Roberts et al, (1987)^44^	29	Linac 6 MeV	9‐12	4.0‐5.0	37‐50	21‐28	Yes	RTOG 0/5	RTOG 2/5 (40%)^a^	RTOG 0/5		RTOG 3/5 (60%)^a^		RTOG 7/24 (29%)^a^	RTOG 8/24 (33%)^a^	RTOG 10/24 (42%)^a^			all eyes within caudal aspect of field
Thrall et al, (1983)^31^	21	Orthovoltage 250 kVp	5‐11		26‐55	28	No												
MacEwen et al, (1977)^15^	43	Orthovoltage 140 kVp	6	6.5	39	14	No												
Madewell et al, (1976)^14^	49	Cobalt‐60 teletherapy 60 Orthovoltage 280 kVp	9‐10	4‐4.5	36‐45	21	No												

Abbreviations: 3DCRT, 3‐dimensional conformal radiation therapy; MV, megavolt; na, not applicable; nd, not described.

Numbers presented as "X/?" in the table can not be further specified from the original publication. They may not represent the true amount of toxicity seen in all cases, as they seem to represent only a fraction of all cases.

Gray columns: high‐grade, grade 3 or 4 acute and late toxicities.

^a^ Toxicity not graded in publication, toxicity grade estimated from description.

^b^ Different scores attributed to same patient (hence >100% of side effects)

## 3 EVOLVING RADIOTHERAPY TECHNIQUES FOR SINONASAL TUMORS IN DOGS

Historically, sinonasal tumors were treated with orthovoltage, cobalt‐60 teletherapy sources and early linear accelerators (linacs), where anatomical landmarks or skin marks were used to set up a radiation field.^5^, ^18^, ^29^, ^30^, ^31^, ^32^, ^33^ In addition, sporadic megavoltage portal films helped in position localization. While easy to perform and inexpensive, the quality of megavoltage portal films is poor and a limiting factor for accurate therapy.^34^ To still provide an acceptable treatment accuracy, radiation oncologists had to counteract the resulting larger setup errors with larger PTV margins of up to 10 mm.^35^ Large PTV margins around the tumor, however, result in large treatment fields and high radiation dose levels to normal tissues.^35^

**Figure 2:**
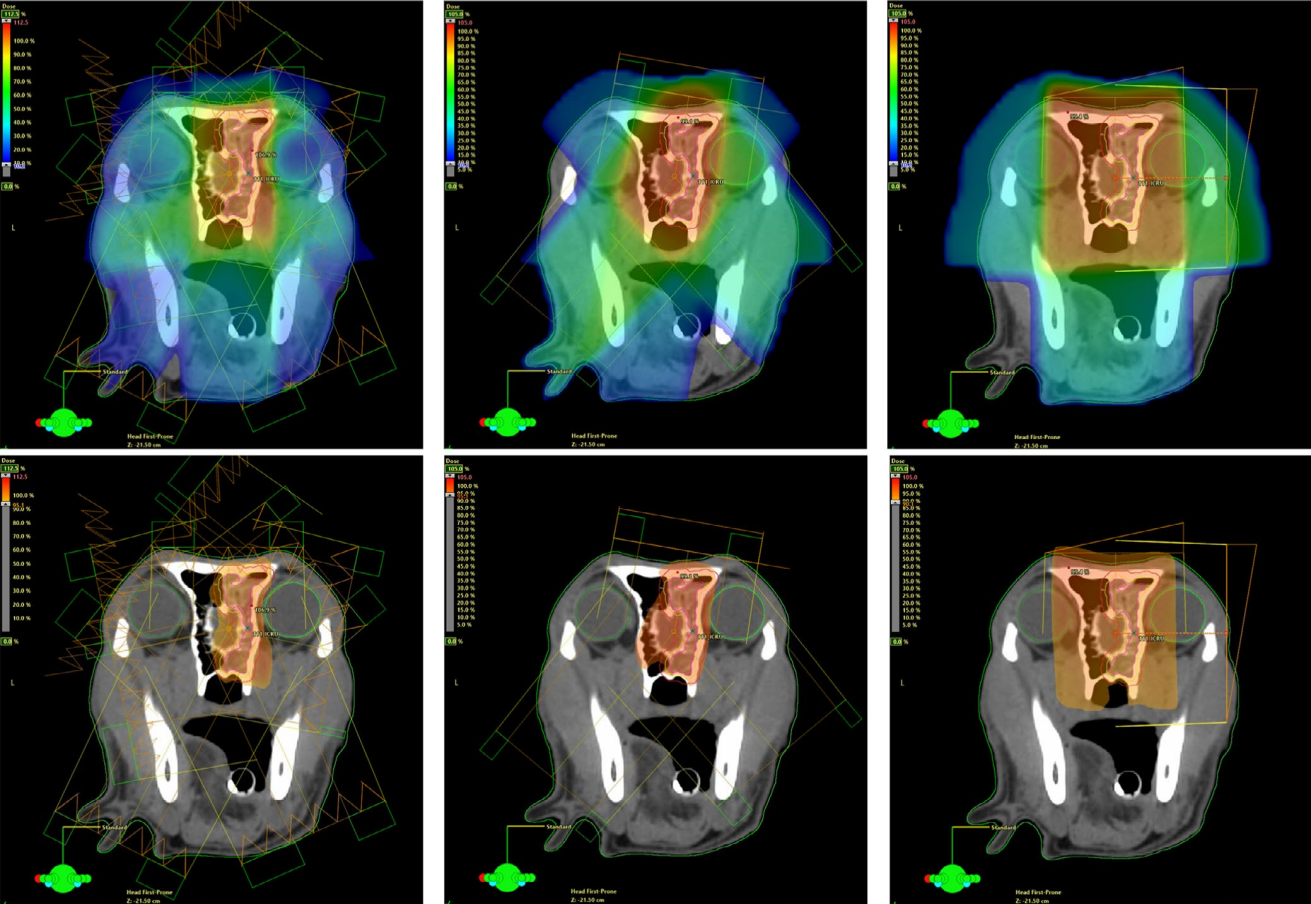
Dose distribution of a treatment plan for a sinonasal tumor at the level of the eyes: Doses in color wash: blue = low doses, green = intermediate doses, orange and red = high doses (dose at prescription). Left side: IMRT plan (7 fields), middle: 3DCRT plan (3 fields), right side: 2D‐RT plan (2 fields). The full dose range is displayed in colors in the top row: 10% to maximum dose; in the bottom row, only the high, clinically relevant tumor dose is displayed: 95% to maximum dose and 90% to maximum dose in 2D‐RT plan, respectively. Depicted organs at risk: green and light green: eyes. Note, how the doses deposited in the ocular structures are very low in the IMRT plan, compared to the 3DCRT and especially the 2D‐RT plan

Furthermore, treatment field arrangements were often simple 2‐dimensional radiation therapy (2D‐RT) and 3‐dimensional conformal photon radiation therapy (3D‐CRT), applied in a single dorsal or in two orthogonal (wedged) fields (Figure 2).^5^, ^18^, ^29^, ^30^, ^31^, ^32^, ^33^ These fields could only be modified with few available parameters, such as beam direction, beam aperture, and the presence of beam shaping modifiers such as lead blocks, wedges, and collimators. Together with the physical properties of the radiation beam, such a simple setup resulted in high doses in healthy tissues surrounding the tumor, inevitably delivering a high dose to ocular structures, hereby causing toxicities.

However, in the last two decades, radiation technology rapidly advanced and the physical accuracy of tumor dose delivery and planning techniques increased tremendously.^36^, ^37^ Treatment delivered by modern linear accelerators (linacs) that have intensity‐modulated radiation therapy (IMRT) or volumetric modulated arc therapy capabilities (VMAT – also referred to as Rapidarc) are produced by sophisticated computer treatment planning programs. These programs utilize complex algorithms to calculate dose distribution delivered via multiple individual beams containing a variation in intensity (or energy fluence) across the entire area of each individual beam. This is achieved with a planning method called inverse treatment planning. In inverse planning the algorithms allow for a more complex array of multiple individual beams of nonuniform intensity, or, in case of Tomotherapy or VMAT, algorithms also allow for delivery of a modulation of the table movement and/ or the gantry.^36^, ^37^, ^38^ The variation in intensity (energy fluence) is created by subdividing the beams into hundreds of beamlets (small beams within the main large beam) of individual intensity levels. As a consequence, the dose distribution from IMRT techniques can conform more tightly to the target. Outside of the target the dose falls off fast, creating a steep dose gradient, which reduces high doses to normal structures (Figure 2) and thus minimizes side effects.^22^, ^39^, ^40^, ^41^ The steep dose gradients, however, are less forgiving for setup errors. To ensure accurate delivery of the dose, daily image guidance has been recommended in dogs treated with IMRT for sinonasal tumors.^35^ The newer treatment machines are often equipped with image guidance capabilities to assure the most precise treatment delivery. Image‐guided radiotherapy (IGRT) makes use of 2D‐ or 3D‐imaging to adjust for positioning errors and target variations or patient's motion during treatment (eg, respiratory or involuntary intestinal). With the use of IGRT during treatment setup, the applied margins for setup errors (eg, PTV margins) can be reduced. In general, the institution's patient position verification and correction strategies determine the extent of the PTV margin for each area of treatment. The margin accounts for errors in patient positioning and inter‐ and intra‐fractional organ variations. As a rule, margins should not be compromised even in case of overlap of a PTV with an adjacent organ at risk. The resulting underdose could endanger the expected tumor control in an unpredictable way.^42^

In veterinary medicine these techniques have been introduced with some delay due to technical and financial limitations. However, some initial publications do show a decreasing severity of side effects associated with tumors located in complex anatomical areas when treated with this advanced technology and planning techniques. Patients treated with IMRT included in two published prospective studies showed only a mild degree of acute conjunctivitis following radiation therapy; however, the overall incidence is increased compared to historical control groups.^22^, ^41^ It is possible that the number of animals suffering from mild acute conjunctivitis was higher in these two studies, simply due to the frequent ophthalmic examinations at regular time points to which the patients enrolled in these prospective studies were submitted (ie, also including those patients that did not show any overt clinical symptoms).^22^, ^41^ A third (retrospective) study reported grade 2‐3 acute ocular side effects in 50% of the dogs.^43^ A decrease in neither acute or late ocular toxicity was reported in this study when comparing patients treated with IMRT to those treated with conventional techniques. The side effects, however, were unilateral and affected usually only the eye ipsilateral to the tumor. Lawrence et al (2010) reported the occurrence of mostly mild late toxicity in 26% of dogs treated with IMRT (prospective assessment of toxicity) compared to 64% of the patients in a historical control group. In this historical group, reported side effects included anterior uveitis, KCS (dry eye syndrome), cataracts, and retinopathies. Most importantly, 56% of the reported side effects in the historical group were graded as severe.^22^ Soukup et al^41^ (2018) reported clinically negligible and self‐limiting acute ocular side effects in 33% of patients, also prospectively assessed for side effects. The differences in fractionation protocols and in the levels of treatment delivery accuracy between the institutions involved in these above mentioned studies might influence the assigned PTV extension margins. Hence, it is impossible to base conclusions on a direct comparison of these results.

## 4 RADIATION‐INDUCED OCULAR SIDE EFFECTS

Radiation induces a variety of changes in the eye and periocular tissues.^7^ Tissue damage depends on total radiation dose, fractionation and, in many structures, on irradiated volume. The dose‐volume parameter is often crucial, the same radiation protocol delivered to a smaller volume (smaller tumor or treatment with higher accuracy) will cause less damage, as less dose is deposited in the organ at risk. Histopathologically, radiation‐induced ocular and periocular damage is well described in the dog.^7^, ^22^, ^30^, ^33^, ^44^ The damage develops along a specific timeline: The side effects (toxicities) are commonly divided into early (acute) and late (chronic) side effects, with the term “late” somewhat arbitrarily defined as >90 days after the start of radiation therapy in most studies^45^ and >30 days in a more recent veterinary study for eyes only.^22^ Early radiation side effects are usually found in tissues with a high proliferative activity: Progressive cell depletion (hypoplasia) and atrophy are accompanied by inflammatory changes. In and around the eye, this leads to blepharitis, keratitis, and damage to the lacrimal glands, leading to KCS. Healing of acute side effects should usually be complete.^45^ In severe cases, however, corneal ulceration, panophthalmitis, and glaucoma can occur, potentially leading to loss of vision or the need for enucleation. Late toxicity consists of various degrees of degenerative changes, caused by parenchymal alterations, connective tissue, and vascular injury. In general, late radiation side effects are irreversible and progressive.^45^ Around the eye, this can include the development of KCS, cataracts, retinopathy, and optic neuropathy, any of which can lead to vision loss.^7^, ^46^ The occurrence of these late side effects was relatively predictable according to one histopathologic study evaluating the ocular side effects of megavoltage irradiation therapy for nasal carcinomas in dogs.^7^ Degenerative angiopathy of retinal vessels with multifocal retinal hemorrhage was first observed around 3‐6 months post‐irradiation and caused slowly progressive retinal degeneration. Cataracts and axonal degeneration in the optic nerve were observed at 6 months and two years after irradiation, respectively. Hence, the authors concluded that the canine eye is sufficiently sensitive to radiation for even relatively low total doses to cause significant long‐term injury.^7^

Table 1 summarizes 36 publications from the existing literature on radiation treatment of sinonasal tumors in dogs. More than half of the publications listed ocular toxicities. Twenty‐four of the publications reported early toxicities, 50% of which reported side effects considered to be severe. Nineteen of the publications reported on late toxicities, 68% of which reported side effects considered to be severe. Most of the studies assessed toxicity retrospectively, whereas prospective assessment of ocular side effects was only found in three studies.^7^, ^22^, ^41^ The proportion of patients in each study considered to have severe side effects ranged from 4%‐60% for early, and 4%‐56% for late toxicity. It was not possible to discern, however, if factors such as tumor size or stage influenced the occurrence or severity of side effects. Furthermore, the majority of authors provided information on the side effects in a descriptive manner rather than along a categorical score: 13/24 (54%) of the early toxicities and 11/19 (58%) of the late toxicities were provided as text only. The remaining 46% and 42%, however, were quantified along scoring systems from human (Toxicity Criteria of the Radiation Therapy Oncology Group (RTOG))^11^ or veterinary medicine (Toxicity Criteria of the Veterinary Radiation Therapy Oncology Group (VRTOG)),^13^ (Tables 2 and 3).

**Table 2:** Early radiation toxicity scoring as proposed by RTOG^11^ and VRTOG^13^

	Grade 0	Grade 1	Grade 2	Grade 3	Grade 4
RTOG^11^	No symptoms	Mild conjunctivitis w/ or w/o scleral injection/ increased tearing	Moderate conjunctivitis w/ or w/o keratitis requiring steroids and/or antibiotics/ dry eye requiring artificial tears/ iritis with photophobia	Severe keratitis with corneal ulceration/ objective decrease in visual acuity or in visual fields/ acute glaucoma/ panophthalmitis	Loss of vision (uni‐ or bilateral)
VRTOG^13^	No changes over baseline	Mild conjunctivitis and/or scleral injection	Keratoconjunctivitis sicca requiring artificial tears, moderate conjunctivitis or iritis necessitating therapy	Severe keratitis with corneal ulceration and/ or loss of vision, glaucoma	

**Table 3:** Late radiation toxicity scoring as proposed by RTOG^11^ and VRTOG^13^

	Grade 0	Grade 1	Grade 2	Grade 3	Grade 4
RTOG^11^	No symptoms	Asymptomatic cataract; minor corneal ulceration or keratitis	Symptomatic cataract; moderate corneal ulceration; minor retinopathy or glaucoma	Severe keratitis; severe retinopathy or detachment	Panophthalmitis/ blindness
VRTOG^13^	No changes over baseline	Asymptomatic cataracts, keratoconjunctivitis sicca	Symptomatic cataracts, keratitis, corneal ulceration, minor retinopathy, mild to moderate glaucoma	Panophthalmitis, blindness, severe glaucoma, retinal detachment	

The following two sections describe and discuss the findings presented in Table 1 in the various ocular and periocular tissues.

### 4.1 Clinical manifestations I: Early ocular toxicity (Table 2)

Eyelids and conjunctiva: Blepharitis as an inflammatory condition of the eyelid with erythema, with or without swelling,^47^, ^48^ occurred in few cohorts of dogs with an incidence of 11.1%^41^ and 45%^8^ after definitive‐intent treatments (applied with rather small doses per fraction), and in 35.7%^8^ of patients after palliative (hypofractionated, large dose per fraction) treatment. Conjunctivitis was reported more frequently ranging from 27% to 78% in multiple studies of dogs, treated with smaller and larger doses per fraction.^7^, ^8^, ^22^, ^33^ This side effect was observed near the end of therapy in some dogs^33^ or much later, occurring up to 4 weeks after therapy in others.^7^ Radiation‐induced conjunctivitis is caused by radiation‐induced hypoplasia of the basal epithelial stem cell layer.^49^ While the epithelial lining will reconstitute itself over time, additional trauma such as mechanical stress or secondary infections due to loss of epithelial barrier function will aggravate the side effects.^45^

Lacrimal gland system: Radiation damage to the (very sensitive) exocrine tear glands reduces tear volume, with the consequence of inducing KCS.^50^, ^51^, ^52^, ^53^ KCS represents a grade 2 early radiation toxicity and was found in 1.8%‐45% of dogs. Again, the dogs in different studies were treated with different types of intent (palliative‐ or definitive‐intent protocols). Hence, a variety of protocols, total doses and different fraction sizes, including smaller fractions, but also hypofractionated and even stereotactic, severely hypofractionated protocols were used.^8^, ^20^, ^30^, ^43^, ^54^, ^55^, ^56^, ^57^ While not available in the veterinary literature, there is a known dose‐response relationship for the toxicity to the lacrimal glandular system in humans^46^, ^58^, ^59^, ^60^: In one study, no patients developed acute toxicities higher than grade 1 as long as the dose to the gland was less than 15Gy (delivered in 2Gy‐fractions). For every 1Gy increase in dose, the probability of higher grade toxicity increased by 23% and the grade of toxicity also increased, if the volume of the gland receiving the specific dose increased.^58^ Furthermore, the observed incidence significantly increases above a threshold dose of 30Gy (delivered in 2Gy‐fractions).^46^, ^58^

Cornea: Keratitis was apparent in 3%‐70% of the dogs,^7^, ^8^, ^33^, ^43^, ^54^, ^56^, ^57^, ^61^ with ulcerative keratitis in 13.5%‐25%.^8^, ^33^ Full thickness corneal ulceration, caused by radiation‐induced corneal epithelial or stromal cell hypoplasia or untreated KCS was described in up to 27% of dogs. Again, the dogs in different studies were treated with a variety of protocols, total doses and different fraction sizes, some protocols even including chemotherapy.^8^, ^33^, ^43^, ^56^, ^57^, ^62^ In humans, doses between 30 and 50 Gy (delivered in small, 2Gy‐fractions) have caused punctate corneal epithelial erosions and at doses of 40‐50 Gy corneal edema may develop.^46^

Lens, retina, and optic nerve: Tissues with a low cell turnover like lens and optic nerve will not manifest early toxicity. Early changes to the retina are also very rare and dose‐dependent. In some dogs, retinal degeneration started early and 14.3% showed a tapetal atrophy after less than 4 weeks post radiation. Again, these changes were observed with different fraction sizes and total doses.^7^

### 4.2 Clinical manifestations II: Late ocular toxicity (Table 3)

Since tumor progression in sinonasal tumor patients following radiation is often seen fairly early post‐treatment, only 29% of dogs are still free of progression at 1 year and reported median survival times do not exceed 10.8‐19.7 months.^5^, ^6^, ^18^, ^19^, ^20^, ^21^, ^22^, ^23^, ^24^ As a result, patients may not live long enough for clinical development of late toxicities to be observed. The rate of late ocular toxicities therefore may be underestimated in the veterinary literature and may actually increase if, in the future, longer patient survival times are achieved with improved treatment success.

Eyelids, conjunctiva, lacrimal gland system, and cornea: According to Jeganathan et al^46^ (2011), radiation side effects include damage to the Meibomian glands and serous acinar lacrimal glands, and squamous metaplasia and keratinization of the palpebral conjunctiva, which can lead to permanent dysfunction of these structures and contribute to KCS. Since direct confirmation of such Meibomian gland, lacrimal gland and conjunctival pathologies requires post‐mortem evaluations or tissue biopsies, they are not assessed on a routine basis in veterinary patients and therefore not likely reported. In the human literature, early KCS is differentiated from late KCS. Early KCS is associated with acute inflammation of the eyelids and conjunctiva as well as apoptotic death of serous acinar lacrimal gland cells resulting in an altered tear film composition and decreased tear film stability and/or volume. As long as sufficient stem cells survive, this is repairable and may not lead to late toxicity. Late effects are associated with eyelid, conjunctival and tear gland fibrosis, conjunctival keratinization, vascular damage and damage to stem cells and are thus permanent. The prevalence of such late effects increases with increasing radiation energy doses.^46^, ^58^, ^59^, ^60^

The prevalence of such side effects is likely underestimated in the veterinary literature, since clinical assessment of post‐treatment KCS in dogs relied on Schirmer tear testing (STT) and documentation of overt keratitis by nonophthalmologists in most reported studies. Late eyelid lesions and conjunctivitis were described to occur in 5%‐13% of patients^8^, ^55^ and persistent KCS in 7%‐22% of irradiated dogs.^8^, ^20^, ^33^, ^54^, ^61^, ^63^ However, the risk of underestimating clinically relevant disease should be decreased by the use of detailed examination and scoring systems as described by Lawrence et al^22^ (2010), by our group^41^ (Tables S1 and S2, respectively)^64^ and by Eaton et al^65^ (2017). These examination and scoring systems include the combined use of some or all of STT, tear film break‐up time (TFBUT), fluorescein and rose bengal stain and corneal sensitivity testing and should be sensitive enough in the hands of veterinary ophthalmologists to detect indirect signs of tear film, ocular surface, and eyelid pathology.

Corneal ulceration as a typical late effect is caused by inadequate corneal protection and regeneration as a result of lack of good quality tear production, trauma from fibrotic changes in the conjunctiva and eyelid margins and excessive epithelial (stem cell) loss.^66^ Clinically, this painful condition often requires extensive ophthalmologic management or enucleation, as the radiation‐induced variant will not heal easily. Up to 56% of dogs were described to have lost one or both eyes due to radiation toxicity (albeit more so in earlier studies).^5^, ^22^, ^29^, ^33^, ^44^, ^61^, ^63^ A 50% risk of ocular loss after 5 years (TD50/5) has been described in humans with a 50 Gy dose (delivered in 2 Gy‐fractions) and a 5% risk (TD5/5) with a 30 Gy dose.^46^

**Figure 3:**
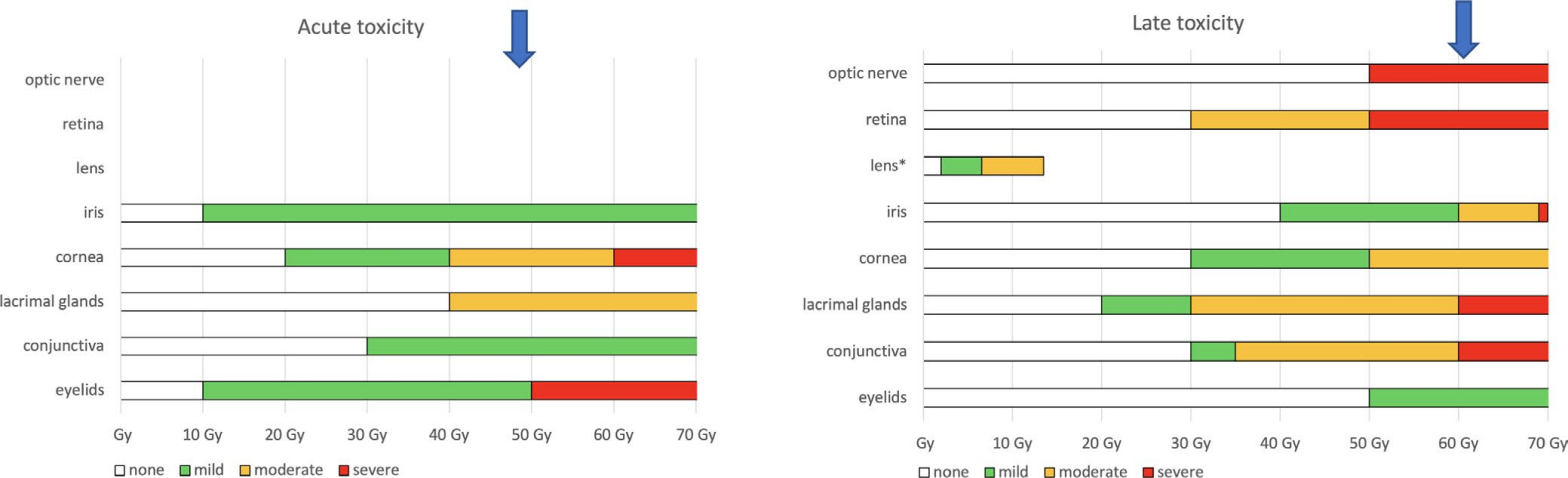
Dose dependencies found in humans according to Jeganathan et al^46^ (2011). The doses represent total doses applied in 2 Gy fractions. Acute (left graph) and late (right graph) toxicity of each individual ocular structure is depicted in color depending on severity: white if absent, green if mild, yellow if moderate, and red if severe. The association with total organ dose is shown on the x‐axis. In order to compare the depicted doses (applied in standard 2 Gy fractions) to differently fractionated protocols (as used in veterinary medicine), the higher fractions can be recalculated into EQD2 (equivalent dose in 2‐Gy fractions) with the formula EQD2 = D*(d+(alpha/beta))/(2 Gy+(alpha/beta)); where D is the total dose, d is the dose per fraction and alpha/beta is a factor quantifying fractionation sensitivity of tissues.^85^ As an example, a commonly used radiation therapy protocol of 10 x 4.2 Gy was recalculated and expected potential acute (alpha/beta = 10) and late side effects (alpha/beta = 2.9; corneal injury) are indicated by blue arrows on the x‐axis. The amount of damage decreases in only partially irradiated organs or with only partial doses

Lens: In the early stage, radiation‐induced cataracts can be distinguished from age‐related cataract by appearing as a dot‐shaped opacity, developing with further progression into a typical donut‐like shape. This morphology may be unique to humans as there are no similar reports in dogs.^67^ The typical late radiation toxicity of the lens is a slow process, albeit also dose‐dependent (Figure 3). The latent time of cataract formation is inversely related to dose and ranges from 6 months to several years (decades in humans).^45^ Most of the dogs are affected over time, because low doses of a few Gy of fractionated irradiation can already induce cataract formation. Reported patient numbers range from 8% to 77%.^5^, ^7^, ^8^, ^22^, ^24^, ^30^, ^33^, ^54^, ^55^, ^68^, ^69^

Retina: Hemorrhages and retinal degeneration occur due to progressive, irreversible degenerative microangiopathy.^70^ Various associated pathologies (retinopathy, tapetal atrophy, hemorrhages, degenerative vasculopathy, ganglion cell degeneration) are reported in 2%‐53% of dogs.^5^, ^7^, ^8^, ^20^, ^22^, ^33^, ^41^, ^63^ In humans, the threshold dose for retinal pathology appears to be around 30‐35 Gy (delivered in 2 Gy‐fractions), but retinopathy has been reported after doses as low as 11 Gy. The TD5/5 is 45‐50 Gy and fraction size also appears to be an important factor: The risk for retinal pathology is significantly increased with fractions above 1.9Gy.^46^, ^71^

Optic nerve: Radiation‐induced optic neuropathy (RION) is a rare side effect of radiotherapy in human patients, described to manifest between 3 months and 8 years after treatment.^46^ In dogs treated with different types of intent (palliative‐ or definitive‐intent protocols), the occurrence is time‐dependent with 13%‐35%^7^, ^22^ and 50%^7^ at 6 months and 2 years, respectively. These numbers, however, only represent the fraction of treated dogs that either received low enough doses to spare the anterior ocular structures or lived long enough to express late effects to the optic nerve.

### 4.3 Proposed management

Optimal management of radiation‐induced ocular side effects includes collaborative care by radiation oncologists and ophthalmologists.^46^ Baseline and repeated ophthalmic examinations have been described and specifically used for dogs undergoing radiation therapy of sinonasal tumors.^22^, ^41^ In general, healing of early radiation effects is based on stem cells that survive within the irradiated volume or migrate from the periphery into the lesion. The higher the administered irradiation doses are, the longer healing of clinically manifested side effects will take. Supportive care generally consists of topical tear stimulation, for example, cyclosporine, lubricant support, vitamin A ointment, or artificial tear substitute therapy and/or topical anti‐inflammatory drugs which can ameliorate ocular symptoms and bridge the time gap until early radiation‐induced ocular toxicity has subsided.^41^, ^55^ Topical antibiotics should be reserved for cases with evidence of secondary bacterial infections. Oral treatment with nonsteroidal or steroidal anti‐inflammatory drugs and stronger pain medications can be administered as needed. Surgical options exist for corneal ulcers^72^ and cataracts.^73^ The success rates for cataract surgery in human patients with radiation‐induced cataracts are good, and the incidence of complications is not higher in these cases compared to routine cataract surgery cases.^74^ However, radiation‐induced collateral damage to other parts of the eye usually makes dogs with radiation‐induced cataracts poor candidates for cataract surgery. Successful treatment approaches in dogs with loss of vision due to radiation‐induced retinal or optic nerve damage (RION, late toxicity) have also not been described. The use of intravitreal anti‐VEGF therapies has shown promise for the treatment of neovascular disease as a result of radiation‐induced ischemia in human patients.^46^ While these humanized antibodies work well in humans, dogs (and other species) rapidly develop antibodies to them. Therefore, such antibodies are often tolerated for only a few doses.^75^

## 5 REVIEW OF OCULAR DOSE‐VOLUME DATA IN DOGS

Ocular side effects were described and graded using either the RTOG scoring system in 1 of 26 (4%) or the VRTOG scoring system in 9 of 26 (35%) articles listed in Table 1. We retrospectively assigned toxicity scores corresponding to the description of toxicities per patient (marked with an asterisks “*” in Table 1) for the papers that did not apply any scoring system. These numbers, however, may lead to uncertain conclusions, since several toxicities can occur in the same patient (even in the same organ). Also, a scoring system can be used in multiple ways: It can pick up every side effect or report only the most severe score (eg, worst toxicity) per patient and organ. Not all authors used the scoring systems in the same way: Some listed only the highest toxicity, some also the lower toxicities evolving over time.

Only two studies have provided the actual radiation doses (dose volumes) that resulted in the reported toxicities.^22^, ^41^ Often, only parts of organs at risk receive a high irradiation dose, specifically in case of overlap with the PTV. However, complete reporting including volume, fraction size, and total dose, such as occasionally provided in the human literature,^58^ would be appropriate and could correlate to a risk probability for developing side effects.^76^ Once the critical dose volumes are known for a certain OAR, a risk probability can be anticipated, possibly yielding a dose‐cut off (such as dose‐volume or maximum dose, or TD5/5).^77^, ^78^ These values should then represent an acceptable grade of toxicity and can be implemented in treatment planning to provide a safe treatment for patients.

RTOG/VRTOG late grade 1 toxicities can already be problematic, since chronic KCS can pose a long‐term treatment challenge and “asymptomatic” cataracts can eventually become vision impairing as radiation‐induced cataracts can be progressive in nature.^46^, ^74^ Late grade 2 toxicities also include a number of pathologies that have the potential to cause impairment or loss of vision and/or loss of the eye. Specifically, “symptomatic” cataracts are vision impairing and can cause lens‐induced uveitis and glaucoma, which tend to cause irreversible loss of vision and chronic pain necessitating enucleation for palliative reasons.^46^, ^79^ Eyes with nonhealing radiation‐induced corneal ulcers might need surgical treatment or enucleation for palliative reasons as well.^44^, ^46^, ^61^, ^80^

Therefore, attempts to minimize grade 1 and 2 toxicities (without compromising tumor control!) are highly recommended. It should, however, be made clear to owners that a certain level of side effects might have to be accepted as a trade‐off for effective treatment of a life‐limiting disease.

Every attempt to avoid late grade 3 VRTOG and grade 3 + 4 RTOG toxicities should be made whenever possible (eg, without compromising tumor control in a definitive‐intent setting), as these typically lead to irreversible vision loss or complete loss of the eye. From the summarized publications of the last three decades, we can learn that a mean dose of 39 Gy to large portions or the whole volume of the eye (given in 10 x 4.2 Gy fractions) will lead to loss of functionality in more than 50% of eyes (grade 3‐4 toxicity), while mean doses <30 Gy seem to preserve functionality.^22^ No further information could be found in the literature for higher or lower total doses, or more or less fraction numbers. These crude numbers can be viewed as a start. However, as technology and medical care improve, which will hopefully lead to longer patient survival and tumor control, we may start noticing a higher incidence of later observed side effects with greater severity and complications.

## 6 CONCLUSION AND FUTURE DIRECTIONS

As with all medical interventions, it is important to find the balance between efficacy and side effects, as some treatments are so toxic that they can become more detrimental than beneficial to the patient.^81^ In general, owners perceive their dogs’ quality of life during and after radiation therapy as good. In one study, 92% of the owners reported to be happy to have elected radiation for their dog's cancer treatment once the course was completed. In that same study, 88% of owners said that they would treat another pet again, if indicated.^82^ With the new technologies and the goal of increasing tumor control, radiation oncologists may be tempted to further increase doses to tumors in the future. Hence, appropriate and standardized reporting of side effects in sinonasal radiation therapy in dogs must become an integral part of patient assessment, especially in clinical trials.

The incoherent use of toxicity scoring systems represents the crux of the matter for gaining further knowledge on tolerated doses in eyes.^83^ Based on the above, the advantage of presenting side effects in a descriptive manner can be appreciated. In publications using scoring systems such as the VRTOG or RTOG scales, several possible tissue changes are summarized into a single score which makes it impossible to discern which tissues were most severely affected. This could be circumvented by using more detailed scoring systems, such as those used by Lawrence et al (2010)^22^ and Soukup et al^41^ (2018), or as found in Eaton et al^65^ (2017). Authors should provide both descriptive and scored (ordinal) information on toxicity and provide information regarding the time points at which side effects were recorded (eg, reported side effects assessed at set time points versus reporting only maximal side effects regardless of time). We therefore propose to use detailed ophthalmic side effect scoring systems such as those described and published by Lawrence et al (2010)^22^ and Soukup et al^41^ (2018) (Tables S1 and S2, respectively),^64^ or the multifunctional clinical scoring system described and illustrated in great detail by Eaton et al^65^ (2017), in addition to established early and late radiation toxicity scoring systems such as the RTOG or VRTOG scales (Tables 2 and 3). The use of such detailed ophthalmic scoring systems should increase consistency across examiners but would require a veterinary ophthalmologist to perform the examinations. In reality, appropriate and consistent future scoring may require a working group including radiation oncologists and ophthalmologists to reach a consensus regarding a useful and practically applicable clinical scoring system. Furthermore, appropriate reporting and prescription of radiation doses as proposed for 3DCRT^84^ and IMRT^42^ are key to the correct interpretation and comparison of treatment outcomes.

Thus, together with descriptions and grading of clinical changes, irradiation dose, volume and fractionation data should be reported as recommended for IMRT.^42^

The technique of IMRT will help to reduce the risk of disabling treatment‐induced toxicities. In case of acceptable toxicities, IMRT might even allow sinonasal tumor treatment with higher doses, which could potentially lead to better tumor control. In order to better use inverse planning, however, we need to know the dose‐volume constraints for organs at risk in dogs. Without knowledge of the dose‐volume constraints of the respective OAR, it is difficult for the planner to know which organs to prioritize for protection during treatment planning. In treatment planning, dose‐volume constraints represent a cut‐off, a safeguard for the respective organ to develop toxicity at a low incidence of <5% or <10%. Early toxicity is not of great relevance, as long as it is self‐limiting, symptomatic treatment is possible and as long as it does not lead to severe painful or late sequelae. Late toxicity, which is typically irreversible, progressive, and untreatable, remains of great concern and should be avoided as much as possible.

## CONFLICT OF INTERESTS

None.

## Supporting information

Table S1

Table S2
