# Supplementary material for: Ocular and periocular radiation toxicity in dogs treated for sinonasal tumors: A critical review
Source: Vet Ophthalmol. 2020 Apr 12;23(4):596–610. doi: 10.1111/vop.12761 (PMC7496316; doi:10.1111/vop.12761)
Supplement: Supplementary file 1 — Table S1 [file VOP-23-596-s001.docx]

**Acute and late ocular toxicity scoring system as proposed by and adapted from Lawrence et al. (2010)^22^**

| **Clinical symptoms** | **Grade** |
| --- | --- |
| Conjunctivitis and/or blepharitis (conjunctival hyperemia and/ or blepharospasm) | 0  1  2  3  4 |
| Keratitis  (presence of lipid corneal degeneration, corneal edema, scarring, pigmentation and/or corneal vascularization; (considered keratoconjunctivitis if associated with conjunctivitis) | 0  1  2  3  4 |
| Ulcerative kerato-conjunctivitis with or without blepharitis (characterized by fluorescein stain retention) | 0  1  2  3  4 |
| Keratoconjunctivitis sicca  (Schirmer tear test <10mm/minute and compatible clinical signs) | 0  1  2  3  4 |
| Anterior uveitis  (ocular vascular injection, aqueous flare/ cell, miosis, or intraocular pressure below normal) | 0  1  2  3  4 |
| Presumed radiation-induced cataracts  (progressive opacification of the lens compared to that noted before radiation therapy) | 0  1  2  3  4 |
| Retinopathy/optic neuropathy  (retinal hemorrhages, detachment, degeneration, or optic nerve damage) | 0  1  2  3  4 |
|  |  |
| Acute toxicity | Total |
| Late toxicity | Total |

All ocular examinations, including slit-lamp biomicroscopy, indirect ophthalmoscopy, Schirmer tear testing, fluorescein and Rose Bengal staining, and applanation tonometry are performed by a Board-certified veterinary ophthalmologist.

Changes are classified as acute if they occur within 1 month of completion of radiotherapy and late if they occur after this time.

All ocular changes are qualitatively scored from 0-4; grade 0 indicates no abnormalities, grade 1 indicates changes only visible to an experienced ophthalmologist/observer using magnification and bright focal illumination, grade 2 represents mild, grade 3 moderate, and grade 4 maximally severe changes.
Apart from grade, ocular changes are classified into the above tabulated clinical syndromes. The first three (1-3) are mutually exclusive and the score that is assigned is the most severe complication manifested in either eye during the entire period of follow-up.
