# Supplementary material for: Ocular and periocular radiation toxicity in dogs treated for sinonasal tumors: A critical review
Source: Vet Ophthalmol. 2020 Apr 12;23(4):596–610. doi: 10.1111/vop.12761 (PMC7496316; doi:10.1111/vop.12761)
Supplement: Supplementary file 2 — Table S2 [file VOP-23-596-s002.docx]

**Ophthalmic examination protocol according to Soukup et al. (2018)^41^**

**First RT Treatment: _________**

**Examination: pre-RT** ☐ **post-RT** ☐ **Number of weeks post RT**:

**Examiner: KV** ☐ **SP** ☐ **Signature:** _

**Date of examination:** _________

**Method:**

- Slitlamp Biomicroscopy (Kowa SL-17, 10x)

- Rebound Tonometry (Tonovet, three low variance measurements: record lowest measurement)

- STT

- Fluorescein

0 = No area of fluorescein staining.

1 = 1 to 25% area of fluorescein staining.

2 = 26 to 50% area of fluorescein staining.

3 = 51 to 75% area of fluorescein staining.

4 = 75 to 100% area of fluorescein staining.

- Rose Bengal (scoring as for fluorescein staining)

- TFBUT (directly after fluorescein application, prior to rinsing excess fluorescein out of eye: let blink to create even tear film, then open, wait and count until first discontinuities in tear film appear)

- corneal sensitivity with cotton tipped applicator (lightly touch central cornea 3x and note number of responses)

- Tropicamide dilatation

- Slitlamp Biomicroscopy of lens (Kowa SL-17, 10x)

- Indirect Ophthalmoscopy (20, 2.2D lens) post-dilatation.

Photos: exterior ocular structures ☐

fundus ☐

**OD OS**

**Tonometry: mmHg mmHg**

**STT: mm/min mm/min**

**TFBUT: sec sec**

**Fluorescein:** 0☐ 1☐ 2☐ 3☐ 4☐ 0☐ 1☐ 2☐ 3☐ 4☐

**Rose Bengal:** 0☐ 1☐ 2☐ 3☐ 4☐ 0☐ 1☐ 2☐ 3☐ 4☐

**Corneal sensitivity:**

**Footnotes exam:**

1. Corneal opacities (edema, infiltrates, deposits, fibrosis, pigmentation and neovascularization) need to be marked and drawn in the schedule below.
2. Lens: location (capsular, cortical, nuclear) and extension (punctate, incipient, incomplete, complete, resorbing) need to be marked and drawn in the schedule below.
3. Retinal detachments and areas of retinal degeneration: the location (central, mid-peripheral, peripheral, nasal, temporal, inferior, superior) needs to be marked and drawn in the schedule below.
4. Retinal tears, hemorrhages and chorioretinitis lesions: the location (central, mid-peripheral, peripheral, nasal, temporal, inferior, superior) and size (in # of optic nerve diameters (OND)) need to be marked and drawn in the schedule below.

**Right eye (OD) Left eye (OS)**


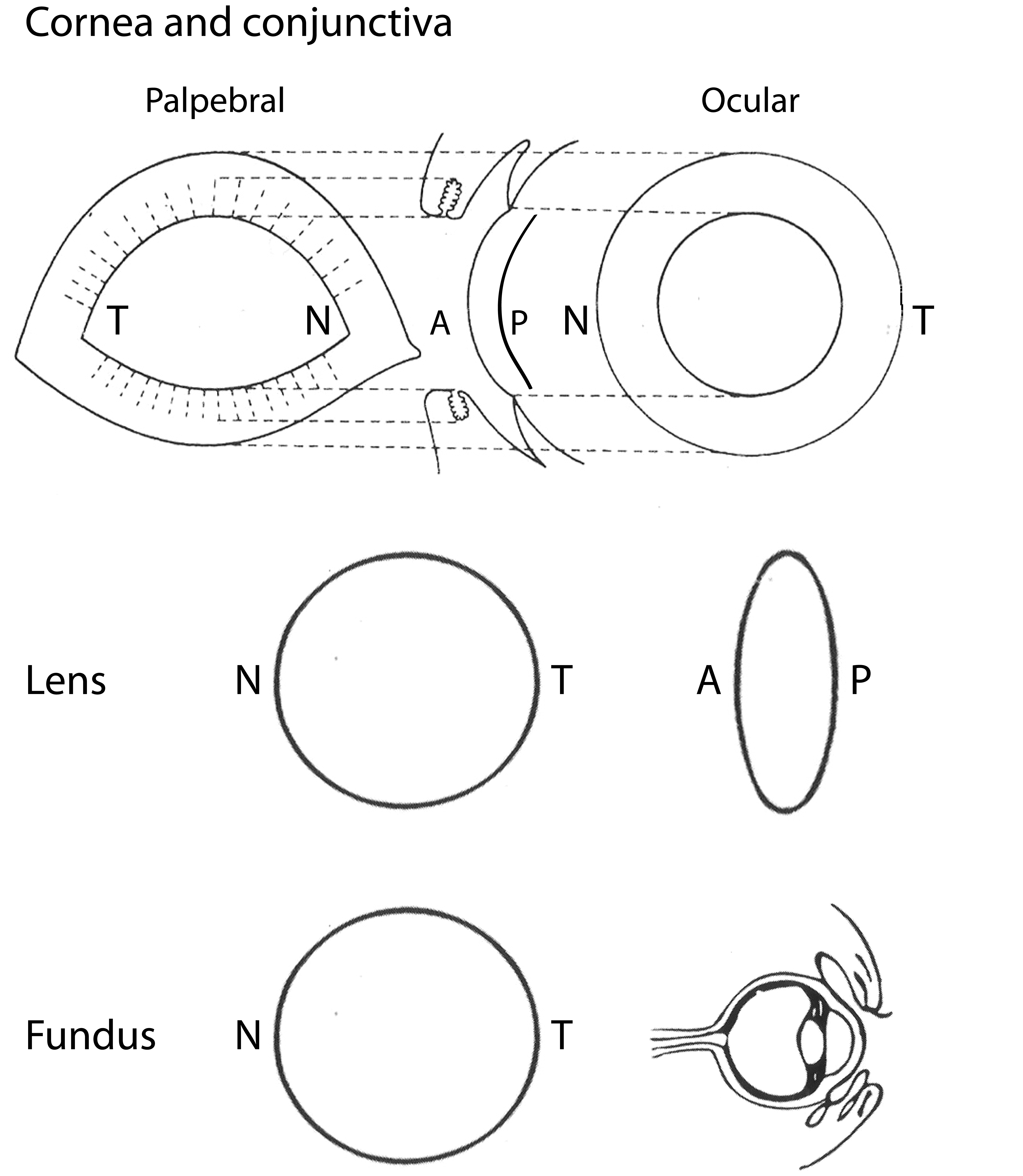


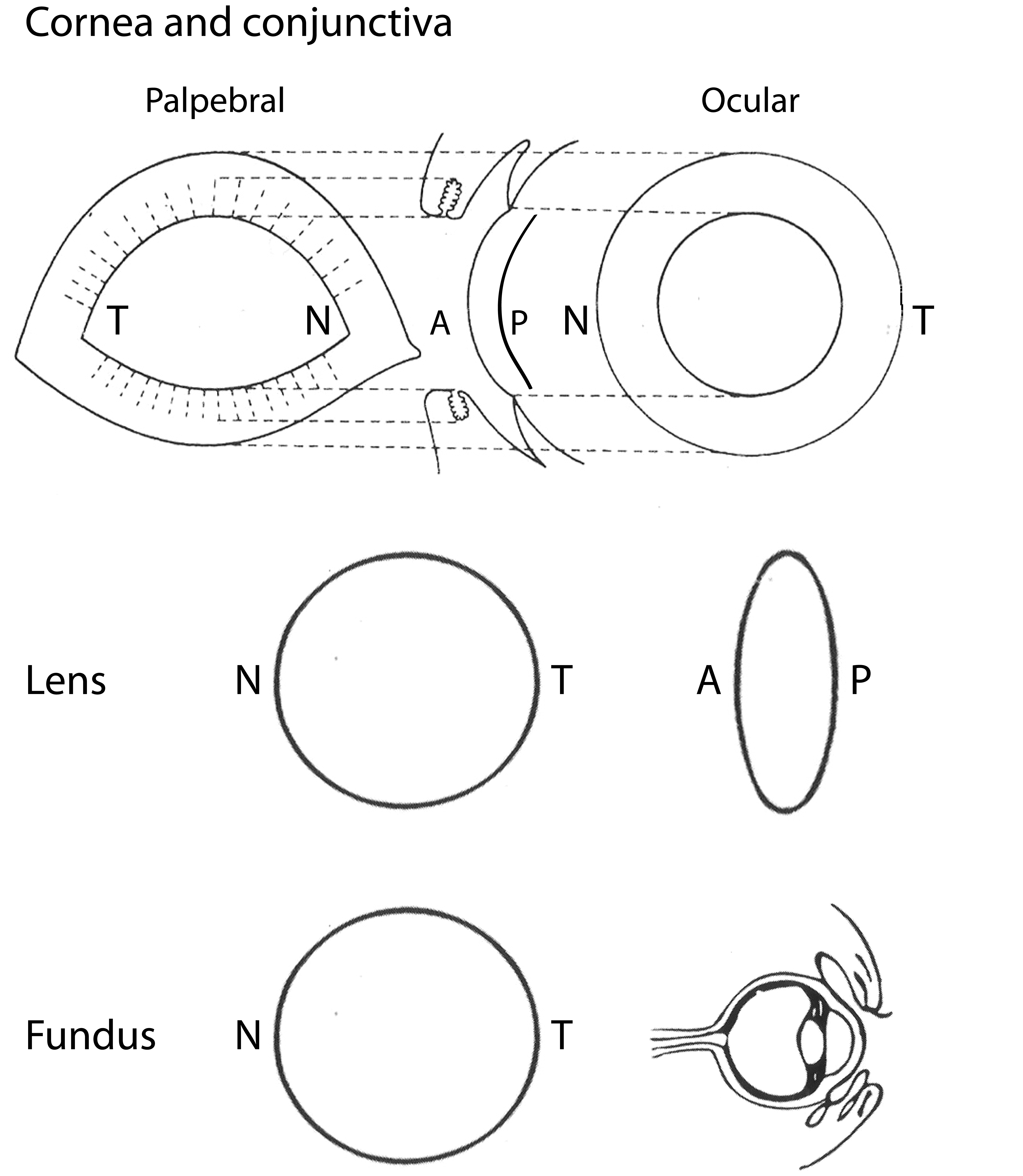


Size: ____________________________

Extent: ____________________________

Size: ONOND____________________________

Location: ____________________________

Location: ____________________________

Location: ____________________________

Location: ____________________________

Extent: ____________________________

1. Menace response: OD: 0☐ 1☐ 2☐ OS: 0☐ 1☐ 2☐
2. Dazzle reflex: OD: 0☐ 1☐ 2☐ OS: 0☐ 1☐ 2☐
3. Palpebral reflex: OD: 0☐ 1☐ 2☐ OS: 0☐ 1☐ 2☐
4. Pupillary light reflex direct: OD: 0☐ 1☐ 2☐ OS: 0☐ 1☐ 2☐
5. Pupillary light reflex indirect: OD: 0☐ 1☐ 2☐ OS: 0☐ 1☐ 2☐
6. Conjuctival congestion: OD: 0☐ 1☐ 2☐ OS: 0☐ 1☐ 2☐
7. Conjuctival chemosis: OD: 0☐ 1☐ 2☐ OS: 0☐ 1☐ 2☐
8. Conjuctival discharge: OD: 0☐ 1☐ 2☐ 3☐ 4☐ OS: 0☐ 1☐ 2☐ 3☐ 4☐
9. Corneal opacity: OD: 0☐ 1☐ 2☐ 3☐ 4☐ OS: 0☐ 1☐ 2☐ 3☐ 4☐
10. Area of corneal opacity: OD: 0☐ 1☐ 2☐ 3☐ 4☐ OS: 0☐ 1☐ 2☐ 3☐ 4☐
11. Corneal pigmentation: OD: 0☐ 1☐ 2☐ 3☐ 4☐ OS: 0☐ 1☐ 2☐ 3☐ 4☐
12. Corneal neovascularization: OD: 0☐ 1☐ 2☐ OS: 0☐ 1☐ 2☐
13. Anterior chamber flare: OD: 0☐ trace☐ 1☐ 2-3☐ 4☐ OS: 0☐ trace☐ 1☐ 2-3☐ 4☐
14. Aqueous cell: OD: 0☐ trace☐ 1☐ 2-3☐ 4☐ OS: 0☐ trace☐ 1☐ 2-3☐ 4☐
15. Cell color: OD: white☐ red☐ brown☐ mix☐OS: white☐ red☐ brown☐ mix☐
16. Iris: OD: 0☐ 1☐ 2☐ 3☐ OS: 0☐ 1☐ 2☐ 3☐
17. Lens: OD: 0☐ 1☐ OS: 0☐ 1☐
18. Vitreal cell: OD: 0☐ trace☐ 1☐ 2-3☐ 4☐ OS: 0☐ trace☐ 1☐ 2-3☐ 4☐
19. Cell color: OD: white☐ red☐ brown☐ mix☐OS: white☐ red☐ brown☐ mix☐
20. Vitreal degeneration: OD: 0☐ 1☐ 2☐ OS: 0☐ 1☐ 2☐
21. Vitreal hemorrhage: OD: 0☐ 1☐ 2☐ OS: 0☐ 1☐ 2☐
22. Retinal detachment: OD: 0☐ 1☐ 2☐ 3☐ 4☐ flat☐ bullous☐ transsudate☐ exsudate☐ blood☐ OS : 0☐ 1☐ 2☐ 3☐ 4☐ flat☐ bullous☐ transsudate☐ exsudate☐ blood☐
23. Retinal tears/holes: OD: 0☐ 1☐ OS: 0☐ 1☐
24. Retinal hemorrhages: OD: 0☐ 1☐ OS: 0☐ 1☐
25. Chorioretinitis lesions: OD: 0☐ 1☐ Active☐ Inactive☐ OS: 0☐ 1☐ Active☐ Inactive☐
26. Retinal vessel attenuation: OD: 0☐ 1☐ 2☐ OS: 0☐ 1☐ 2☐
27. Optic nerve atrophy: OD: 0☐ 1☐ OS: 0☐ 1☐
28. Optic nerve cupping: OD: 0☐ 1☐ OS: 0☐ 1☐
29. Retinal degeneration: OD: 0☐ 1☐ 2☐ 3☐ OS: 0☐ 1☐ 2☐ 3☐

**Ophthalmic examination grading system based on modified MacDonald-Shadduck System (Altmann et al. (2010)^75^)**

**Reflexes**

0 = Positive

1 = Negative

2 = Slow or incomplete

**Conjunctival congestion**

0 = Normal.

1 = Mild.

2 = Intense.

**Conjunctival chemosis/swelling**

0 = Normal.

1 = Mild.

2 = Intense.

**Conjunctival discharge**

0 = Normal, no discharge.

1 = Serous

2 = Mucous

3 = Mucopurulent

4 = Purulent

**Cornea**

0 = Normal cornea. appears with the slitlamp as having a bright grey line on the epithelial surface and a bright grey line on the endothelial surface with a marble-like grey appearance of the stroma.

1 = Some loss of transparency. Only the anterior half of the stroma is involved as observed with an optical section of the slitlamp. The underlying structures (incl iris and lens) are clearly visible with diffuse illumination, although some cloudiness can be readily apparent with diffuse illumination.

2 = Moderate loss of transparency. In addition to involving the anterior stroma, the cloudiness extends all the way to the endothelium, the stroma has lost ist marble-like appearance and is homogeneously white, as observed with an optical section of the slitlamp. With diffuse illumination underlying structures are clearly visible, there is some loss of iris detail, however.

3 = Marked loss of transparency. Involvement of the entire thickness of the stroma. With optical section, the endothelial surface and deeper structures are still visible to the extent that the observer is still able to grade flare (barely and only if significant!), iritis, observe for lenticular changes and pupillary responses. With diffuse illumination the deeper structures (excluding the pupillary aperture) are no longer visible.

4 = Complete loss of transparency. With optical section the endothelium and deeper structures are no longer clearly discernable. The cloudiness removes the capability to grade flare, iritis, observe for lenticular changes and pupillary responses. With diffuse illumination the deeper structures are no longer visible.

**% Area of corneal opacity**

0 = Normal cornea with no area of cloudiness.

1 = 1 to 25% area of stromal cloudiness.

2 = 26 to 50% area of stromal cloudiness.

3 = 51 to 75% area of stromal cloudiness.

4 = 75 to 100% area of stromal cloudiness.

**Corneal pigmentation**

0 = Normal cornea with no area of pigmentation.

1 = 1 to 25% area of pigmentation.

2 = 26 to 50% area of pigmentation.

3 = 51 to 75% area of pigmentation.

4 = 75 to 100% area of pigmentation.

**Corneal neovascularization**

0 = No corneal vascularization

1 = Vascularization is present but vessels have not invaded the entire corneal circumference. Where localized vessel invasion has occurred, they have not penetrated beyond 2 mm.

2 = Vessels have invaded 2 mm or more around the entire corneal circumference.

**Anterior chamber/aqueous flare**

0 = No protein is visible in the anterior chamber when viewed by an experienced observer using slit-lamp biomicroscopy; a small, bright, focal slit-beam of white light; and high magnification.

Trace = Trace amount of protein is detectable in the anterior chamber. This protein is only visible with careful scrutiny by an experienced observer using slit-lamp biomicroscopy; a small, bright, focal slit-beam of white light; and high magnification.

1+ = Mild amount of protein is detectable in the anterior chamber. The presence of protein is immediately apparent to an experienced observer using slit-lamp biomicroscopy and high magnification. Such protein is detected only with careful observation with the naked eye and a small, bright, focal slit-beam of white light.

2-3+ = Moderate amount of protein is detectable in the anterior chamber. These grades are similar to 1+, but the opacity would be readily visible to the naked eye of an observer using any source of a focused beam of white light. This is a continuum of moderate opacification with 2+ being less apparent than 3+.

4+ = Large (severe) amount of protein is detectable in the anterior chamber. Similar to 3+ but the density of the protein approaches that of the lens. Additionally, frank fibrin deposition is frequently seen in acute circumstances. It needs to be noted that because fibrin may persist for a period of time after partial or complete restoration of the blood-aqueous barrier, it is possible to have resorbing fibrin present with lower numeric assignations for flare (e.g. 1+ flare with fibrin).

**Aqueous cell**

0 = No cells are seen in a single field of the focused slit lamp beam. No cells are visualized as the slit lamp beam is swept across the anterior chamber.

Trace = Occasional (1-5) cells are seen in a single field of the focused slit lamp beam. When the instrument is held stationary, not every optical section contains circulating cells.

1+ = 6-15 cells are seen in a single field of the focused slit lamp beam. When the instrument is held stationary, each optical section contains circulating cells.

2+ = 16-25 cells are seen in a single field of the focused slit lamp beam. When the instrument is held stationary, each optical section contains circulating cells.

3+ = 26-50 cells are seen in a single field of the focused slit lamp beam. When the instrument is held stationary, each optical section contains circulating cells. Keratic precipitates or cellular deposits on the anterior lens capsule may be present.

4+ = Greater than 50 cells are seen in a single field of the focused slit lamp beam. When the instrument is held stationary, each optical section contains circulating cells. Keratic precipitates or cellular deposits on the anterior lens capsule may be present. Fibrin depositions, hypopion or clumps of cells may persist for some period of time after the active exsudation of cells into the anterior chamber has diminished or ceased entirely. Thus, it is possible to have hypopion present with lower numeric assignations for cell (e.g. 1+ cell with hypopion).

**Cell color**

Aqueous or vitreal cell may be observed as white, red or brown, and will be recorded as one category as follows: predominantly brown (>75% brown), predominantly white (>75% white), predominantly red (>75% red) or mixed (other ratios of white, red and/or brown). Cell color types will not be counted, rather they will be subjectively categorized by the ophthalmologist.

**Iris**

0 = Normal iris without any hyperemia of the iris vessels.

1 = Subtle injection or engorgement of the iridal blood vessels.

2 = Marked injection of the iridal vessels throughout the iris.

3 = iris neovascularization / presence of preiridal fibrovascular membranes (PIFMs).

**Lens**

The lens should be evaluated and graded as either 0 (= normal) or 1 (= abnormal).The presence of lenticular opacities should be described as outlined below and the location noted.

Potential locations include: capsular (anterior and/or posterior), cortical (anterior, posterior, equatorial), nuclear, or any combination of the above. The extent of the opacity will be classified into one of the following categories:

0 = Normal.

1 = Abnormal

*Punctate:* A focal or multifocal, discrete, dot-like lens opacity that is visible only to an experienced observer using slit-lamp biomicroscopy and high magnification.

*Incipient:* A focal lens opacity that is visible upon gross inspection of the eye with an indirect ophthalmoscope or other focal light source and retro-illumination. The view of the fundus is minimally impaired by the opacity. Upon slit-lamp biomicroscopy the opacity can be localized to a specific region of the lens and other regions of the lens appear normal.

*Incomplete:* A diffuse lens opacity that is visible upon gross inspection of the eye with an indirect ophthalmoscope or other focal light source and retro-illumination. The view of the fundus is significantly impaired but a tapetal reflex can still be obtained and portions of the fundus may be visible upon careful indirect ophthalmoscopic examination through a maximally dilated pupil.

*Complete:* A diffuse lens opacity that is visible upon gross inspection of the eye with an indirect ophthalmoscope or other focal light source. The fundus cannot be seen and a tapetal reflex cannot be elicited. Upon slit-lamp biomicroscopy the entire lens is opaque.

*Resorbing:* A diffuse lens opacity visible upon gross inspection of the eye with an indirect ophthalmoscope or other focal light source. The fundus may or may not be visible and a tapetal reflex may or may not be obtainable. The lens capsule may be wrinkled and the lens itself is dehydrated and flattened or liquid and soft in appearance. Upon slit-lamp biomicroscopy the entire lens is involved in the opacity.

.

**Vitreal cell and color**

Vitreal cell scores are assigned by using the same estimate of cells per field (0, 1-5, 6-15, 16-25, 26-50, and >50) as described before for anterior chamber cell scores. Vitreal cell color estimates will be performed as described for anterior chamber cell color estimation.

**Vitreal degeneration**

0 = No degeneration of the vitreous. Some structure (observed as indistinct, white to grey, twisting lines) in the vitreous is normal.

1 = Mild degeneration of the vitreous. Some thicker strands of condensed vitreous and some areas of liquefaction (recognised as darker areas) are present.

2 = Significant degeneration of the vitreous. Clumps and multiple thick strands of condensed vitreous (collagen and cellular material) are present throughout the vitreous, but mostly in the ventral part. The view of the fundus can locally be impaired by the opacities. Liquefied vitreous is recognised as darker areas within the vitreous and because of the lagging, swirling movement displayed by vitreal debris when the eye moves.

**Vitreal hemorrhage**

0 = No vitreal hemorrhage.

1 = Minor hemorrhage present. The view of the fundus is minimally impaired by the opacity.

2 = Significant hemorrhage present, obstructing the observer’s view of the fundus. In the most extreme case the entire vitreal cavity can be filled with blood.

**Retinal detachment**

Retinal detachments should be characterized regarding degree of elevation (flat vs significant elevation), type of subretinal fluid present (transsudate, exsudate, blood) and location within the fundus (central, mid-peripheral, peripheral and nasal, temporal, inferior or superior fundus affected). Detachments will be graded depending on fundus area size affected according to the following schedule:

0 = No retinal detachment present.

1 = Retinal detachment affecting 1-25% of the fundus area.

2 = Retinal detachment affecting 26-50% of the fundus area.

3 = Retinal detachment affecting 51-100% of the fundus area.

4 = Complete, disinserted retinal detachment.

**Retinal tears/holes**

0 = Not present

1 = Present, the lesion size compared to the optic nerve head and the location of the lesion within the fundus (central, mid-peripheral, peripheral and nasal, temporal, inferior or superior fundus affected) will be specified.

**Retinal hemorrhages**

0 = Not present

1 = Present, the size of the lesions will be compared to the size of the optic nerve head and the location of the lesions within the fundus (central, mid-peripheral, peripheral and nasal, temporal, inferior or superior fundus affected) will be specified.

**Retinitis/chorioretinitis lesions**

0 = Not present

1 = Present, the size of the lesions will be compared to the size of the optic nerve head and the location of the lesions within the fundus (central, mid-peripheral, peripheral and nasal, temporal, inferior or superior fundus affected) will be specified. Retinitis/chorioretinitis lesions will also be graded as active or inactive (chorioretinal scars) lesions.

**Retinal vessel attenuation**

0 = Not present

1 = Only present in the periphery

2 = Present in the central and peripheral retina

**Optic nerve atrophy**

0 = Not present

1 = Present

**Optic nerve cupping**

0 = Not present

1 = Present

**Retinal degeneration**

0 = Not present

1 = Retinal degeneration affecting 1-25% of the fundus area.

2 = Retinal degeneration affecting 26-50% of the fundus area.

3 = Retinal degeneration affecting 51-100% of the fundus area.
